# Application of the Suzuki-Miyaura Reaction in the Synthesis of Flavonoids

**DOI:** 10.3390/molecules18044739

**Published:** 2013-04-22

**Authors:** Mamoalosi A. Selepe, Fanie R. Van Heerden

**Affiliations:** School of Chemistry and Physics, University of KwaZulu-Natal, Private Bag X01, Scottsville 3209, South Africa; E-Mail: 208518849@stu.ukzn.ac.za

**Keywords:** Suzuki-Miyaura reaction, flavonoid, chalcone, flavone, isoflavone, neoflavone, 4-arylcoumarin, biflavone

## Abstract

The application of the Suzuki-Miyaura reaction in the synthesis of flavonoids, an important class of natural products, is reviewed. This reaction has not only been employed to provide access to flavonoid nuclei, but has also been applied to the synthesis of dimeric flavonoids and in the synthesis of libraries of flavonoid derivatives for biological activity studies. The classes of flavonoids that are discussed are the chalcones, flavones, isoflavones, neoflavones, biflavones and derivatives of flavonoids obtained by C-C bond formation via the Suzuki-Miyaura reaction.

## 1. Introduction

**Figure 1:**
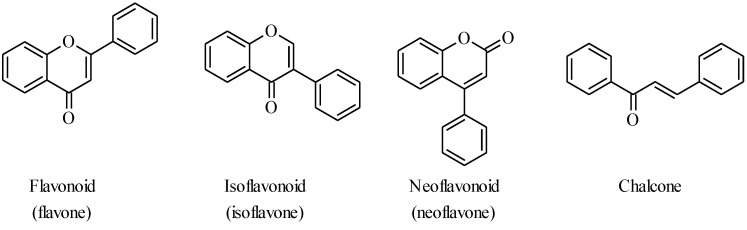
Different classes of flavonoids.

Flavonoids are plant secondary metabolites with a C_6_C_3_C_6_ skeleton and can be divided into three main classes, *i.e.*, flavonoids, isoflavonoids and neoflavonoids (4-arylcoumarins). Chalcones, the biogenetic precursor to flavonoids, are often also classified as flavonoids (Figure 1). Different oxidation states and different substituents contribute to the diversity of flavonoid structures. Flavonoids play an important role in plant physiology and are of interest to humans as a result of biological activities such as antioxidant, anticancer and estrogenic activity of individual flavonoid derivatives.

As a result of the biological activity of flavonoids, there is an interest in the development of synthetic procedures that can conveniently give access to these molecules and their derivatives. One of the methods that has recently been employed successfully in the synthesis of flavonoid scaffolds is the Suzuki-Miyaura reaction. The Suzuki-Miyaura reaction normally involves insertion of palladium into a sp^2^-hybridized C-X bond and consequently the major application of this reaction is in the construction of the flavonoid nucleus of chalcones, flavones, isoflavones and neoflavones rather than in the synthesis of their reduced derivatives. Unlike other methods that have been employed in the synthesis of flavonoids, the Suzuki-Miyaura reaction often employs mild conditions that are compatible with a variety of functional groups. This enables the synthesis of flavonoids of natural origin and derivatives from precursors bearing sensitive substituents [1,2,3,4]. Moreover, the method readily offers access to a variety of flavonoids for biological activity studies by using different organoboron starting materials in the final stages of the synthesis [4,5,6,7,8,9,10]. The method is amenable to large scale synthesis due to the stability and commercial availability of a wide range of boronic acids/esters, and the ease of working up the reaction mixture [6,11,12]. In the following sections, the syntheses of the four classes of flavonoids and some of their dimeric analogues using the Suzuki-Miyaura reaction are reviewed.

## 2. Chalcones

### 2.1. Chalcone Monomers

Chalcones are readily accessible by the Claisen-Schmidt condensation of benzaldehydes with acetophenones [13,14,15,16]. Other routes that have been developed include acylation of phenols with cinnamic acids [17], Heck coupling of aryl iodides with aryl vinyl ketones [18], and palladium-catalyzed coupling of arylpropargyl alcohols with aryl halides [19,20].

**Scheme 1:**
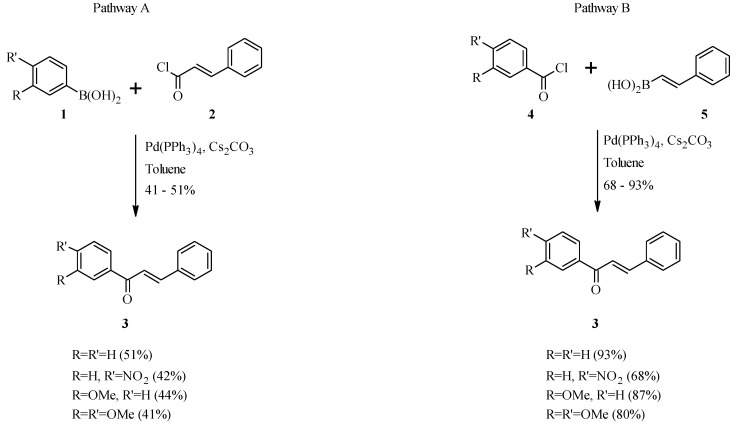
Preparation of chalcones by Eddarir and co-workers [21].

The synthesis of chalcones by the Suzuki-Miyaura reaction was first demonstrated by Eddarir and co-workers in 2006 [21]. Their strategy was based on two pathways; the first one involved coupling of arylboronic acids **1** with cinnamoyl chloride (**2**), whereas the second pathway involved coupling of styrylboronic acid (**5**) with benzoyl chlorides **4** (Scheme 1). Moderate yields (41–51%) of chalcones **3** were obtained from pathway A, when using Haddach and McCarthy’s conditions [anhydrous toluene, Pd(PPh_3_)_4_, Cs_2_CO_3_] [22]. These conditions gave the chalcones in good to excellent yields (68–93%) from pathway B. 

**Scheme 2:**
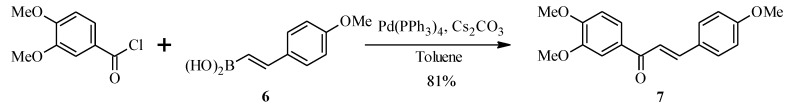
Preparation of a methoxylated chalcone by the coupling of a benzoyl chloride and a styrylboronic acid [21].

**Scheme 3:**
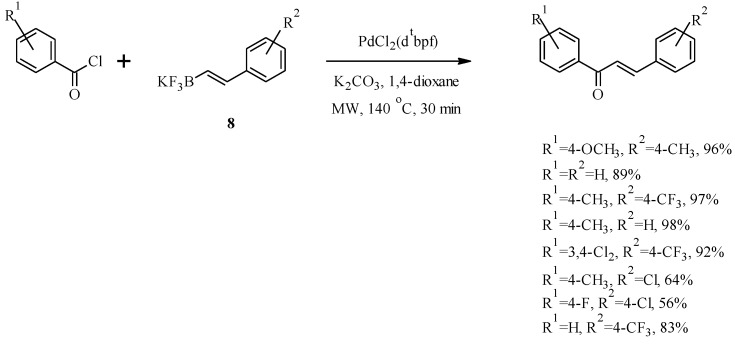
Preparation of chalcones by the coupling of a benzoyl chloride with a potassium styryltrifluoroborates [23].

Since most naturally-occurring chalcones are oxygenated on the aromatic rings, the application of route B was further extended to the synthesis of methoxylated chalcone **7** in a good yield from 3,4-dimethoxybenzoyl chloride and styrylboronic acid **6** (Scheme 2) [21]. Al-Masum *et al.* used a similar approach to prepare a number of unnatural chalcones by using potassium styryltrifluoroborates **8** as the starting material and performing the reaction under microwave conditions (Scheme 3) [23].

**Scheme 4:**
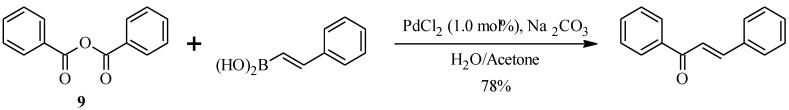
Synthesis of a chalcone by cross-coupling of benzoic anhydride and phenylboronic acid [24].

In 2008, Xin reported the synthesis of aryl ketones by the reaction of arylboronic acids with benzoic anhydride [24]. The reaction was extended to the reaction of styrylboronic acid with benzoic anhydride (**9**) in the presence of PdCl_2_ and Na_2_CO_3_ in H_2_O/acetone (1:1) to give the chalcone in 78% yield (Scheme 4) [24]. However, the functional group tolerance of this method is yet to be tested on chalcones with varying substitution patterns including those of natural origin.

Zuo *et al.* obtained a number of aryl-substituted chalcones by preparing either the aryl-substituted acetophenone or benzaldehyde via the Suzuki-Miyaura reaction, followed by the traditional Claisen-Schmidt synthesis of the chalcones. Most of the synthesized compounds had moderate to strong anticancer activity against five cancer cell lines, and have NF-kB nuclear translocation inhibition activities [25]. Vieira *et al.* used a similar approach by using the Claisien-Schmidt reaction to prepare brominated chalcones which were subjected to the Suzuki-Miyaura reaction to yield arylated chalcones [26]. In this investigation, the emphasis was on developing reaction conditions that are environmentally benign and they introduced the use of the non-toxic polyethyleneglycol as solvent for the reaction.

### 2.2. Bichalcones

**Figure 2:**
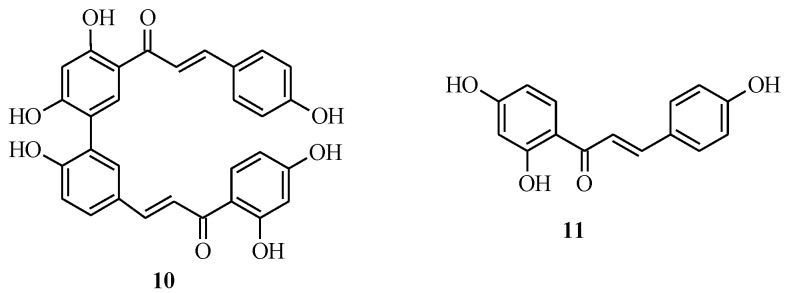
Structures of a rhuschalcone and its monomer.

Bichalcones consist of two chalcone monomers linked by either an ether or a C-C bond. The latter are the most prevalent class of bichalcones, while the former type has been reported from the *Rhus* genus only [6]. Mihigo *et al.* reported the total synthesis of rhuschalcone VI (**10**), an antiplasmodial C-C bridged natural bichalcone which consists of two molecules of isoliquiritigenin (**11**) [6] (Figure 2). The synthetic protocol for **10** was based on the Suzuki reaction for the construction of the C-C biaryl linkage and solvent-free aldol condensation for the synthesis of the chalcone monomers [14].

**Scheme 5:**
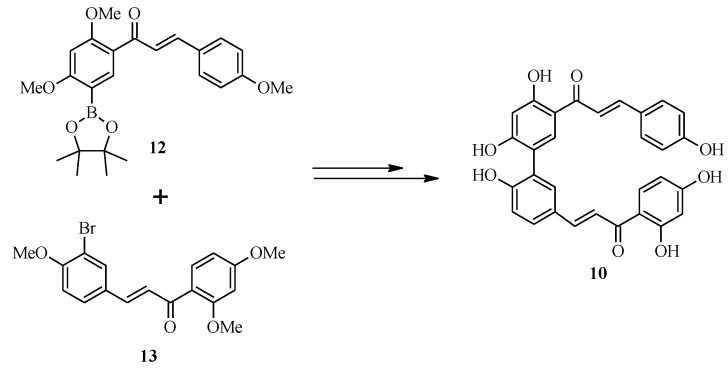
The first proposed synthetic route for rhuschalcone VI (**10**) [6].

Initially, it was envisaged that rhuschalcone VI (**10**) and its derivatives would be prepared by the Suzuki coupling of chalconylboronate ester **12** and bromochalcone **13** followed by demethylation (Scheme 5). However, the synthesis of chalconylboronate esters from bromochalcones by lithium-halogen exchange and reaction of the lithiated species with 2-isopropoxy-4,4,5,5-tetramethyl-1,3,2-dioxaborolane proved to be challenging. Thus, the reaction sequence was changed [6].

**Scheme 6:**
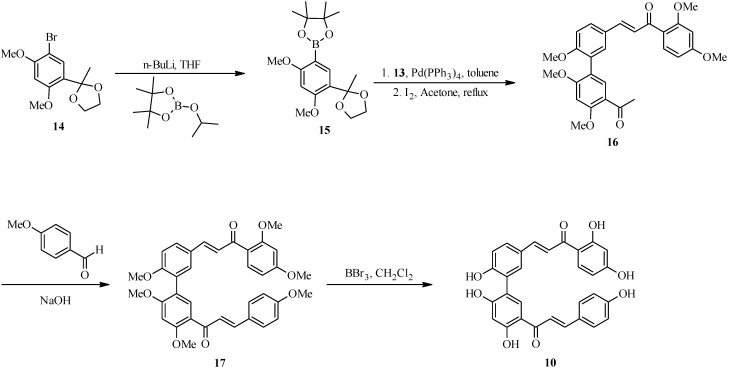
Alternative route for the synthesis of rhuschalcone VI (**10**).

In the modified route (Scheme 6), a boronate ester **15** was formed in the early stages from protected bromoacetophenone **14**. Coupling of **15** with bromochalcone **13** in the presence of Pd(PPh_3_)_4_ and subsequent acetyl deprotection gave arylated chalcone **16**. Solvent-free Claisen-Schmidt condensation of **16** with *p*-methoxybenzaldehyde gave a dimeric chalcone **17**, which was completely or partially demethylated to give rhuschalcone VI (**10**) and its analogues [14]. This synthetic strategy was further expanded to the synthesis of other unnatural arylated chalcones for structure-activity relationship studies, by reaction between boronate ester **15** and bromochalcones with different substitution patterns [6].

## 3. Flavones

### 3.1. Flavone Monomers

Classical approaches to the synthesis of flavones are the Baker-Venkataraman rearrangement, followed by cyclization of the resulting β-diketone [27,28,29], the Allan-Robinson [30] and the Algar-Flynn-Oyamada methods [31,32]. Although other classes of flavonoids have been successfully prepared by the Suzuki-Miyaura reaction, there are not many reports on the synthesis of the flavone nucleus by this method. The main challenge with this procedure is the difficulty of accessing the prerequisite precursors for the Suzuki-Miyaura reaction, which are the 2-halochromones.

**Scheme 7:**
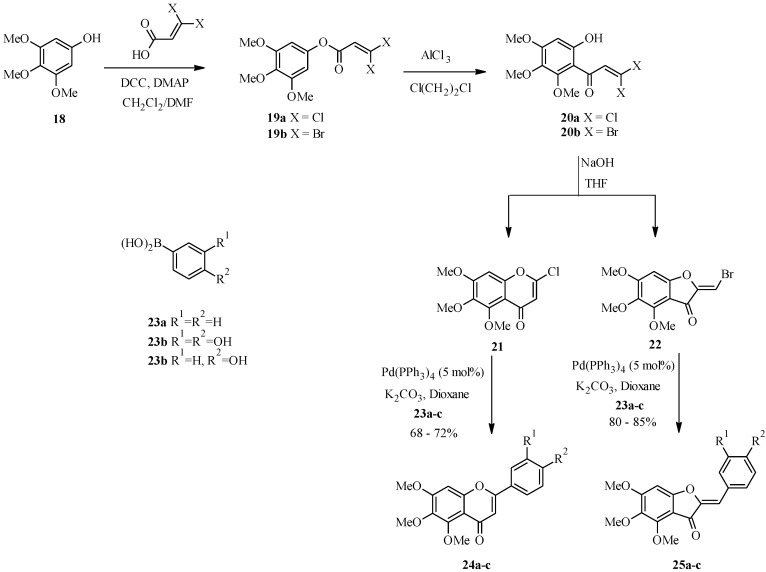
Preparation of flavones and aurones by the Suzuki-Miyaura reaction [33].

Kraus and Gupta reported the synthesis of flavones by the palladium-catalyzed coupling of 2-chlorochromone **21** and arylboronic acids **23a**–**c** (Scheme 7) [33]. Even though the Suzuki-Miyaura reaction is normally conducted with bromides and iodides, it is noteworthy that the reaction of 2-chlorochromone **21** with boronic acids under standard Suzuki conditions gave good yields of the corresponding flavones **24a**–**c** (68–72%). 2-Chlorochromone **21** was in turn prepared from a sequence that involved esterification of phenol **18** with 3,3-dichloroacrylic acid, followed by a Fries rearrangement and base-catalyzed cycloelimination of the resulting dichloroketone **19a**. An attempt to prepare a 2-bromochromone from dibromoketone **19b** gave an intermediate **22** which was coupled with boronic acids **23a**–**c** to give aurones **25a**–**c** (Scheme 7).

A number of authors have synthesized flavones by using classical methodologies, but have used the Suzuki-Miyaura reaction to introduce additional aryl or heterocyclic groups onto the flavone skeleton [34,35,36,37,38,39,40,41].

**Figure 3:**
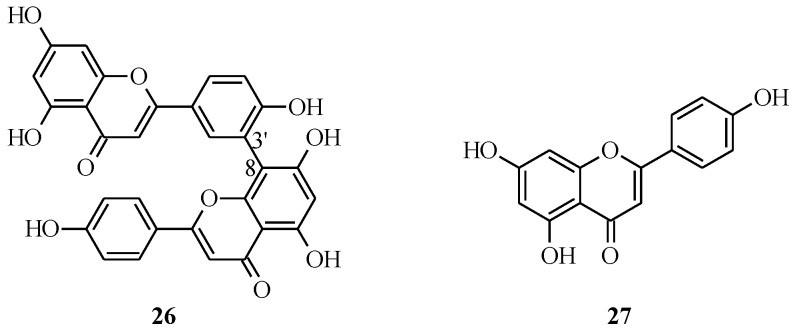
Structures of amentoflavone and its monomer.

Amentoflavone (**26**) consists of two apigenin (**27**) units connected at the C-8 and C-3' positions (Figure 3).

### 3.2. Biflavones

The methods that have been employed for the synthesis of C-C linked dimeric flavones are the Ullmann reaction [42] and metal-catalyzed cross-coupling reactions such as the Stille and Suzuki-Miyaura reactions [43,44,45]. The application of the Suzuki-Miyaura reaction to the synthesis of biflavones was first demonstrated by Muller and Fluery in the synthesis of amentoflavone (**26**) and its methyl derivatives [44].

**Scheme 8:**
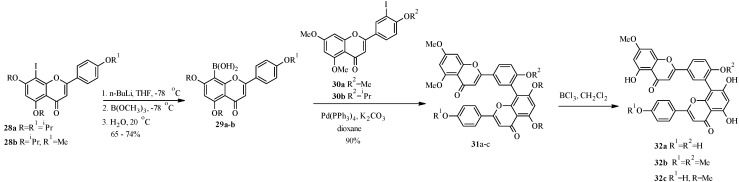
Preparation of derivatives of amentoflavone derivatives [44].

Two synthetic strategies were explored for the synthesis of derivatives **31** and **32** of **26**. The first route involved coupling of 8-flavonylboronic acids **29a** or **29b** with 3'-iodoflavones **30a** and **30b** and subsequent cleavage of the protecting groups (Scheme 8) [44]. The 8-flavonylboronic acids **29a** and **29b** were prepared by lithiation of 8-iodoflavones **28a** and **28b**, respectively, followed by quenching of the lithiated species with triisopropyl borate and aqueous work up.

**Scheme 9:**
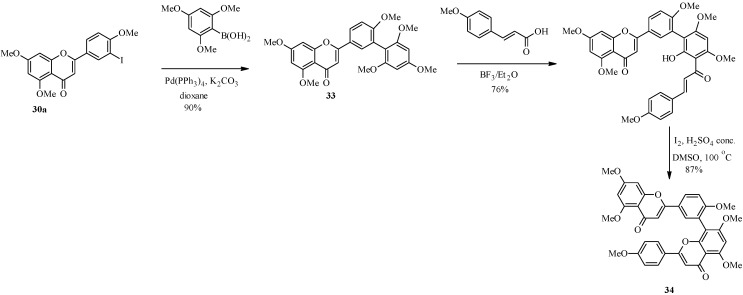
Second route for the preparation of amentoflavone derivatives [44].

The second route proceeded by coupling of 3'-iodoflavone **30a** with 2,4,6-trimethoxyphenylboronic acid to give the arylated flavone **33** (Scheme 9). The second flavone ring was constructed by acylation of **33** with *p*-methoxycinnamic acid, followed by oxidative cyclization. This gave a naturally-occurring hexamethyl derivative of amentoflavone, dioonflavone (**34**) [44].

**Scheme 10:**
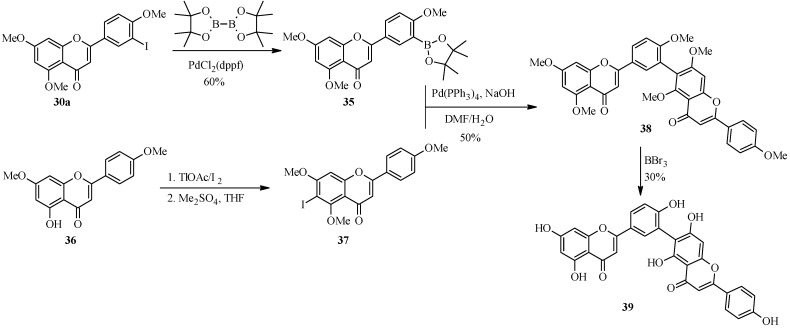
Preparation of robustaflavone (**39**) [45].

In 1998, Zembower and Zhang prepared an inhibitor of the hepatitis B virus, robustaflavone (**39**) (Scheme 10) [45]. Robustaflavone (**39**) differs from amentoflavone (**26**) in the connection between the apigenin monomers which are between C-6 and C-3' instead of C-8 and C-3'.

The key steps in the synthesis of robustaflavone (**39**) were TlOAc-mediated C-6 iodination of apigenin derivate **36** to give the 6-iodoflavone **37** and synthesis of the 3'-flavonylboronate (**35**) by palladium-catalyzed cross-coupling of 3'-iodoflavone 30a with bis(pinacolato)diboron (Scheme 10) [45]. An attempt to convert the 3'-iodoflavone **30a** into the corresponding boronic acid by the procedure employed in the synthesis of amentoflavone (**26**) was unsuccessful. The two precursors were coupled under standard Suzuki conditions to give **38**, which was demethylated with BBr_3_ to give the target compound robustaflavone (**39**).

**Scheme 11:**
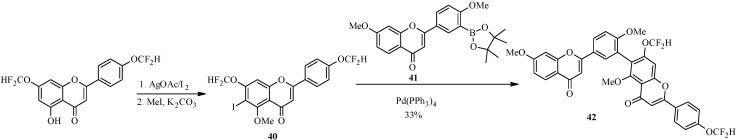
Preparation of *gem*-difluoromethylenated biflavones.

The synthesis of C-C linked biflavones by the Suzuki-Miyaura protocol has also been extended to the preparation of unnatural biflavonoids. An example is the *gem*-difluoromethylenated biflavone **42**, which was prepared by Zheng and co-workers by the palladium-catalyzed coupling of 3'-flavonylboronate ester **41** with 6-iodoflavone **40** (Scheme 11) [46]. In this case the regioselective iodination at position 6 was achieved by using AgOAc/I_2_ instead of the more toxic TlOAc.

**Figure 4:**
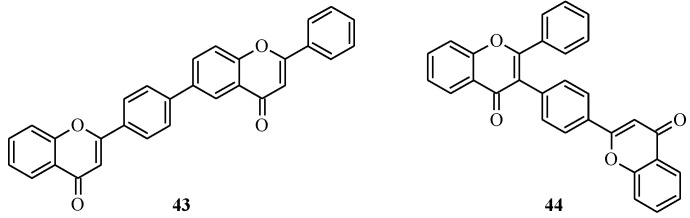
Structures of two unsubstituted biflavones.

More recently, the unnatural biflavones **43** and **44** were prepared by the Suzuki-Miyaura reaction of appropriately functionalized flavones using Pd(PPh_3_)_4_ as a catalyst [43]. The biflavones **43** and **44** together with other unnatural biflavones synthesized by the Stille coupling reaction were tested for inhibition of group II secretory phospholipase A_2_ (_S_PLA_2_IIA) and their inhibitory activity compared with that of the natural biflavones amentoflavone (**26**) and ochnaflavone. Of the active compounds, **44** (Figure 4) was also found to exhibit inhibitory potency comparable to that of amentoflavone (**26**) whereas **43** (Figure 4) exhibited a weaker activity [43].

## 4. Isoflavones

### 4.1. Isoflavones

There are two long-established procedures for the preparation of isoflavones which are still widely used, *i.e.*, the deoxybenzoin route and the chalcone route [47,48,49]. Other methods which have been developed include reductive cleavage of isoxazoles, intramolecular ketene cycloaddition followed by decarboxylation, rearrangement and cyclisation of chalcone epoxides and rearrangement of flavanones [49,50]. The methods that have been developed more recently are the Wacker-Cook tandem conversion of α-methylene deoxybenzoins into isoflavones [51] and the Cu(I)-mediated cyclization of 3-(2-bromophenyl)-3-oxopropanal [52]. Regardless of the many new synthetic approaches presented, the application of many of them has not been demonstrated in the synthesis of polyhydroxylated isoflavones and isoflavones bearing other naturally-occurring substitution patterns.

**Scheme 12:**
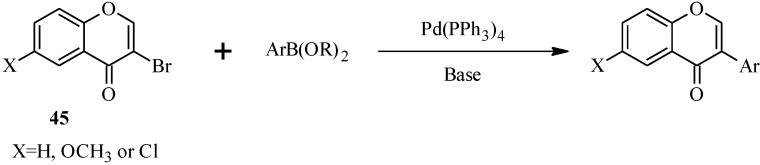
Preparation of isoflavones by the Suzuki reaction [53].

In 1988, Suzuki and co-workers were the first to demonstrate the versatility of the palladium-catalyzed cross-coupling reactions in the synthesis of isoflavones from 3-bromochromones **45** and arylboronic acids/esters (Scheme 12) [53]. Following this, the Suzuki-Miyaura cross-coupling reaction has been applied in several instances to the synthesis of isoflavones in the presence of palladium(0) or palladium(II) catalysts [3,11,12,54,55]. Examples of catalysts which have successfully facilitated this C-C bond formation are Pd(PPh_3_)_4_[1,53], (C) [11,12], *trans*-[PdCl_2_(2-ethyl-2-oxazoline-к^1^*N*)_2_] [55], Pd(dppf)_2_Cl_2_ [3,56], benzothiazole-oxime-based Pd(II) catalyst [54], and Pd(OAc)_2_ in the presence of poly(ethyleneglycol) [56,57] or 2-(2,6-dimethoxybiphenyl)dicyclohexylphosphane (SPhos) [4].

**Scheme 13:**
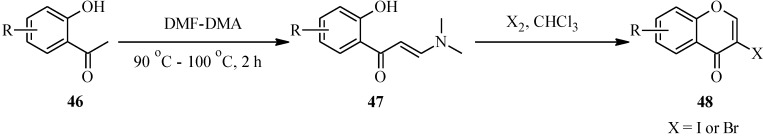
Preparation of 3-halochromones by Gammill’s protocol [58].

Unlike the 2-halochromone derivatives required in the synthesis of flavones by the Suzuki reaction, the 3-halochromone precursors for isoflavones can be conveniently prepared by Gammill’s protocol [58], which involves condensation of appropriately substituted 2'-hydroxyacetophenones **46** with DMF-DMA to form enaminoketones **47**. Halogen-mediated ring closure of the enaminoketones gives the corresponding 3-halochromones **48** [58] (Scheme 13). Alternatively, the 3-halochromones can be obtained by direct halogenation of chromones [4].

**Scheme 14:**
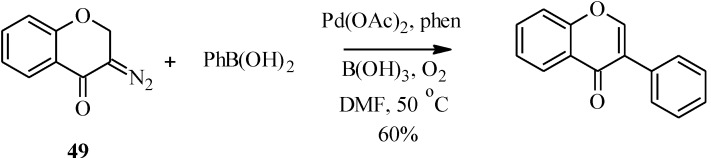
Oxidative cross coupling of phenylboronic acid with a diazochromone [59].

In a variation of the Suzuki-Miyaura reaction, Tsoi *et al.* reported the formation of an unsubstituted isoflavone by the palladium-catalyzed oxidative cross-coupling of a diazochromone (**49**) with phenylboronic acid in 60% yield (Scheme 14) [59].

### 4.2. Preparation of Isoflavone Analogues for Biological Activity Studies

**Scheme 15:**
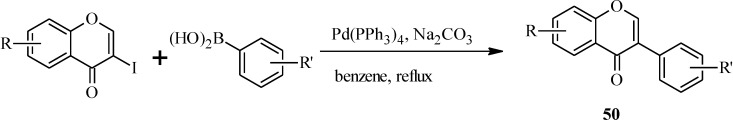
Preparation of a series of isoflavones by Vasselin and co-workers [61].

As a result of the readily availability of 3-halochromones and their potential to be elaborated into a wide range of compounds by coupling with different boronic acids in the final stages of the synthesis, several groups have taken advantage of the Suzuki coupling reaction for the synthesis of libraries of novel compounds based on the isoflavone scaffold for biological activity studies [4,60,61]. For example, Vasselin *et al.* synthesized a series of fluoro, methoxy, nitro and amino isoflavones (**50a**–**o**) from appropriately substituted 3-iodochromones and arylboronic acids (Scheme 15) [61]. However, an attempt to prepare 5,7-dibenzyloxy-3-iodochromone from 4,6-dibenzyloxy-2-hydroxyacetophenone resulted in an inseparable mixture of products while a poor yield (29%) was obtained for 3-iodo-5,7-dimethoxychromone from the corresponding enaminoketone. 

The synthesized isoflavones were tested for *in vitro* growth inhibition of human breast (MDA-MB-468 and MCF-7) and colon (HT29 and HT-116) cancer cell lines. The isoflavones **50d**, **50f**, **50h**, **50k**, **50l** and **50o** showed pronounced growth inhibition of MDA-MD-468 cells co-incubated with TBDD, a powerful inducer of cytochrome P450 (CYP)-1A1 activity. This suggested that the isoflavone derivatives were potential substrates for (CYP)-1A1 bioactivation [61].

**Scheme 16:**
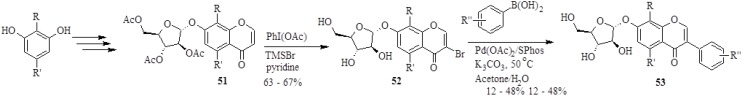
Synthesis of isoflavone glycosides by the Suzuki-Miyaura reaction [4].

In 2008, Wei and Yu synthesized 26 isoflavone glycoside derivatives based on the structure of a potent inhibitor of α-glucosidases of rat liver microsomes, 7-*O*-α-D-arabinofuranosyloxy-4',8-dihydroxyisoflavone (**53a**), also called A-76202 [4]. The analogues of **53a** were prepared by coupling 3-bromochromones **52** to differently substituted arylboronic acids in the presence of Pd(OAc)_2_, SPhos and K_2_CO_3_ in a water-based solvent (H_2_O/acetone) (Scheme 16). However, in certain instances the reaction conditions were slightly altered and coupling was performed in the absence of a ligand, with NaOAc as base and MeOH as solvent to accommodate substrates which were more susceptible to degradation. 

**Table:** 

50	R	R'
**a**	H	4-Cl
**b**	H	4-Br
**c**	H	3-NO_2_
**d**	H	3-OMe
**e**	H	4-OMe
**f**	H	3,4-di-OMe
**g**	6-F	4-OMe
**h**	6-F	3,4-di-OMe
**i**	6-F	3,4-OCH_2_O
**j**	7-F	4-OMe
**k**	7-F	3,4-di-OMe
**l**	8-F	3,4-di-OMe
**m**	6,7-di-F	3,4-di-OMe
**n**	6,8-di-F	3,4-di-OMe
**o**	7,8-di-F	3,4-di-OMe

The 3-bromochromones **52** were in turn prepared by direct bromination of the chromones **51** at C-3 by treatment with PhI(OAc), TMSBr and pyridine [4]. The synthetic natural product **53a** and its derivatives were evaluated for α-glucosidase inhibition [4]. The results showed that the stereochemistry of the α-D-arabinofuranosyl unit and the 8-hydroxy group in the A-ring are essential for the activity, whereas modifications at the B-ring did not adversely affect the α-glucosidase inhibitory activity of the isoflavone 7-*O*-glycosides [4]. It is noteworthy that the 3-OMe and 4-NMe_2 _derivatives (**53e** and **53k**, respectively) were three fold more active than the parent compound **53a** (Scheme 16) [4]. 

**Table:** 

62	R	R'	R''	*IC_50_* (μM) *
**a**	OH	H	4-OH	0.018
**b**	OH	H	H	0.040
**c**	OH	H	4-OMe	0.022
**d**	OH	H	2-OMe	0.015
**e**	OH	H	3-OMe	0.006
**f**	OH	H	4-Me	0.035
**g**	OH	H	2-Me	0.020
**h**	OH	H	3-Me	0.050
**i**	OH	H	3-OH	0.017
**j**	OH	H	4-F	0.015
**k**	OH	H	4-NMe_2_	0.008
**l**	OH	H	4-NHBoc	0.020
**m**	OH	H	4-CF_3_	0.030
**n**	OH	H	4-SiMe_3_	(35% at 0.050)
**o**	H	OH	H	10
**p**	H	OH	4-OMe	8
**q**	H	OH	2-OMe	10
**r**	H	OH	3-OMe	10
**s**	H	OH	4-Me	7
**t**	H	OH	3-Me	5
**u**	H	OH	4-OH	15
**v**	H	OH	3-OH	(33% at 50)

* Inhibition of α-glucosidase.

**Scheme 17:**
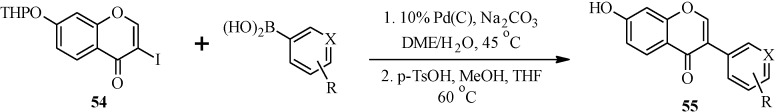
Preparation of isoflavones by Matin and co-workers [5].

Matin and co-workers [5] also prepared a series of isoflavones by coupling of 3-iodochromone **54** and a variety of arylboronic acids in the presence of Pd(C) [11], followed by cleavage of the THP protecting group (Scheme 17). The isoflavones were screened together with other compounds (chalcones, flavones, flavanones, isoflavones and pyrazole derivatives) for dual PPARα and γ agonism. Of the 77 tested compounds, the isoflavones **55a**, **55c**, **55e** and **55i** were identified as novel potent dual PPARα and γ agonists, which could serve as future leads in PPAR-related disorders that include type II diabetes mellitus and metabolic syndrome [5].

**Scheme 18:**
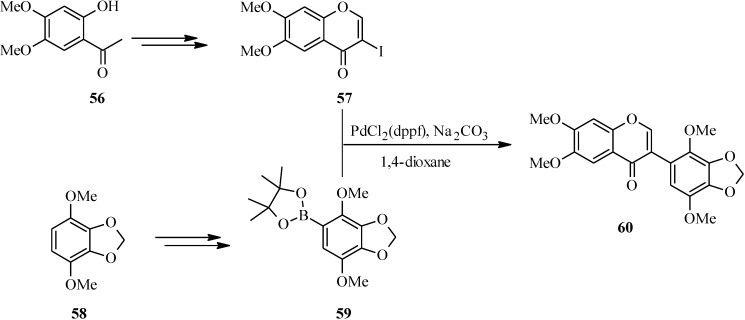
Total synthesis of glaziovianin A (**60**) [62].

In 2007, Kigoshi’s group reported the total synthesis glaziovianin A (**60**), a metabolite of *Astelia glazioviana* that exhibited cytotoxicity against HL-60 cells [60,62]. As illustrated in Scheme 18, the synthesis of glaziovianin A (**60**) commenced by preparing the 3-iodochrome **57** from acetophenone **56** by Gammill’s procedure [58], and the boronic acid **59** from aryl derivative **58**. Coupling of 3-iodochromone **57** with phenylboronic acid **59** gave glaziovianin A (**60**) [60,62].

**Table:** 

55	X	R	55	X	R
**a**	C	3,5-di-OMe	j	C	2-OMe, 3,5-di-F
**b**	C	4-OMe	k	C	3,4,5-tri-F
**c**	C	3,4-OCH_2_O-	l	N	2,4-di-OMe
**d**	C	4-CF_3_	m	C	4-Cl
**e**	C	4-F	n	C	3-F
**f**	C	3,4-di-OMe	o	C	2-OMe
**g**	C	3,4-O(CH_2_)_2_-O	p	C	3-OCF_3_
**h**	C	2,4-di-F	q	C	3-OBn
**i**	C	3-OMe	r	C	3,4,5-tri-OMe

**Scheme 19:**
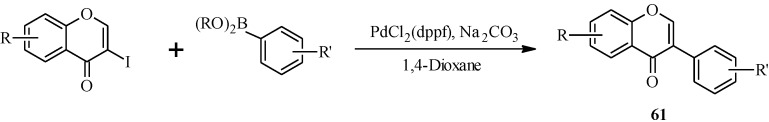
Preparation of glaziovianin A derivatives [60].

Thereafter, they employed the same strategy to prepare glaziovianin analogues **61a**–**i** by altering the substituents on the A- and B-rings (Scheme 19) [60]. The synthesized compounds were tested for cytotoxicity against HeLa S_3_ cells. Of the screened compounds, the 7-*O*-allyl derivative **61i** (IC_50_ = 0.19 μM) was found to be more cytotoxic than the parent compound **60** (IC_50_ = 0.59 μM) [60]. Moreover, **61i** was found to be a more potent M-phase inhibitor [60]. 

**Table:** 

61	R	R'
**a**	6,7-di-OMe	2',5'-di-OMe, 3',4'-OC(CH_3_)_2_O-
**b**	6,7-di-OMe	2',3',4',5'-tetra-OMe
**c**	6,7-di-OMe	3,4-OCH_2_O-
**d**	6-OMe, 7-OTHP	2',5'-di-OMe, 3',4'-OCH_2_O-
**e**	5,6,7-tri-OMe	2',5'-di-OMe, 3',4'-OCH_2_O-
**f**	6-OMe, 7-OH	2',5'-di-OMe, 3',4'-OCH_2_O-
**g**	6-OMe, 7-OBn	2',5'-di-OMe, 3',4'-OCH_2_O-
**h**	6-OMe, 7-*O*-propargyl	2',5'-di-OMe, 3',4'-OCH_2_O-
**i**	6-OMe, 7-*O*-allyl	2',5'-di-OMe, 3',4'-OCH_2_O-

### 4.3. Synthesis of Soy Isoflavones

**Figure 5:**
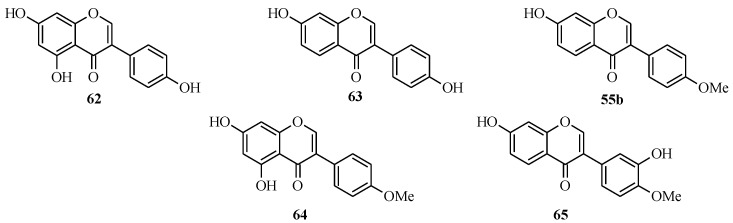
Structures of soy isoflavones.

The soy isoflavones consist of genistein (**62**), daidzein (**63**), formononetin (**55b**), biochanin A (**64**) and the less common glycitein (**65**) [50,57,63,64] (Figure 5). They normally exist as 7-*O*-glycosides, which are metabolized into the aglycones [50,65]. These isoflavones, particularly genistein, are frequently referred to as phytoestrogens, because they are non-steroidal plant-derived compounds with the ability to bind to estrogen receptors and modulate estrogenic responses [57,63,65,66]. The estrogenic properties of the soy isoflavonoids have been extensively studied with regards to health benefits [50,63,65,66,67]. The consumption of phytoestrogen-rich food has been linked to protection against hormone-dependent breast cancer and prostate cancer, alleviation of postmenopausal disorders, osteoporosis as well as cardiovascular protection [50,63,66,67]. 

**Scheme 20:**
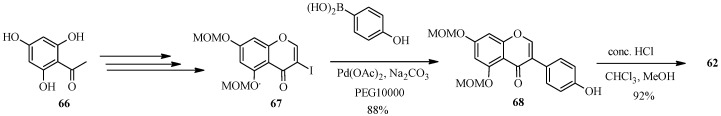
Preparation of genistein (**62**) by Priefer’s group [64].

The synthesis of genistein (**62**) by the Suzuki-Miyaura reaction was first reported in 2010 [2,64], while that of formononetin was reported by Matin and co-workers in 2009 in the preparation of a series of isoflavones for biological activity studies [5]. Priefer’s group synthesized genistein (**62**) in five steps as shown in Scheme 20, which involved preparation of 3-iodo-5,7-dimethoxymethoxychromone (**67**) from phloroacetophenone (**66**) by Gammill’s procedure and coupling of the 3-iodochromone **67** with commercially available 4-hydroxyphenylboronic acid using Pd(OAc)_2_, poly(ethylene glycol) (PEG10000) and Na_2_CO_3_. The MOM-protecting groups on the resulting isoflavone **68** were removed with HCl to give genistein (**62**) [64].

**Scheme 21:**
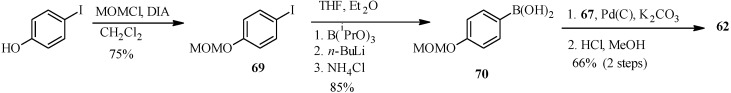
Preparation of genistein (**62**) by Selepe *et al.* [2].

In the same year Selepe *et al.* concurrently reported the synthesis of genistein from the MOM-protected 3-iodochromone **67** (Scheme 21) [2]. Their synthesis involved preparation of the boronic acid **70** from aryl iodide **69** in a one-pot sequence that involved addition of *n*-BuLi to a solution of aryl iodide **69** and triisopropyl borate in THF/Et_2_O (1:2), followed by the hydrolysis of the boronate ester with an NH_4_Cl solution. Heterogeneous Pd(C)-assisted cross-coupling of boronic acid **70** with 3-iodochromone **67**, prepared by Gammill’s protocol and removal of the MOM protecting groups gave genistein (**62**).

**Scheme 22:**
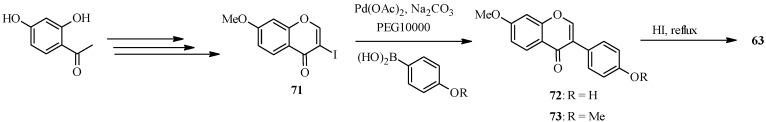
Preparation of daidzein and its methyl derivatives by Priefer’s group [57].

The syntheses of daidzein (**63**) and its methyl derivatives isoformononetin (**72**) and dimethyldaidzein (**73**) were also reported by Priefer’s group under similar conditions to those employed for genistein (**62**) (Scheme 22) [57]. However, the main precursor for the C-C bond formation was 3-iodo-7-methoxychromone (**71**), which was coupled to 4-hydroxyphenylboronic acid and 4-methoxyphenylboronic acid to give isoformononetin (**72**) and dimethyldaidzein (**73**), respectively. Demethylation of **72** and **73** with HI in refluxing chloroform gave daidzein (**63**) [57].

### 4.4. Synthesis of Prenylated Isoflavonoids

Prenylated isoflavonoids are attained by *C*- or *O*-prenylation. Prenylation is often carried out after construction of the isoflavonoid framework [68,69,70]. More complex prenylated isoflavonoids are obtained by cyclization of the prenyl side chains to adjacent hydroxy groups to give furano or pyrano rings, or by modification of phenolic A- and B-rings as well as the C-ring via oxidation or incorporation of other additional substituents [2,3,71,72,73,74]. 

**Figure 6:**
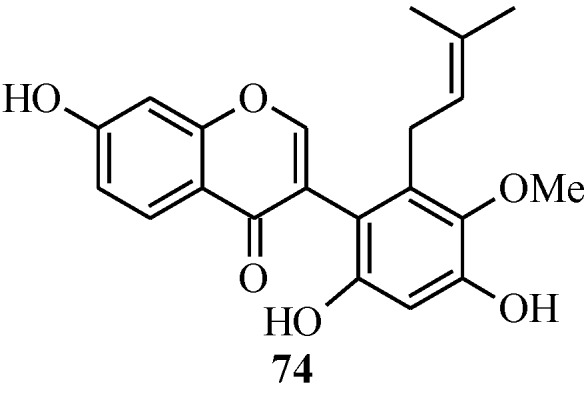
Structure of kwakhurin.

In 2005, Ito and colleagues reported the first total synthesis of kwakhurin (**74**) (Figure 6), a 6'-prenyl-phytoestrogen that was isolated from *Pueraria mirifica* (Leguminosae) [1]. The initial synthetic strategy for **74**, which was based on the deoxybenzoin route, failed to give the targeted compound in the last step that involved deprotection of the isopropyl protecting groups. Thus, an alternative route which enabled the use of easily removable protecting groups was sought [1].

**Scheme 23:**
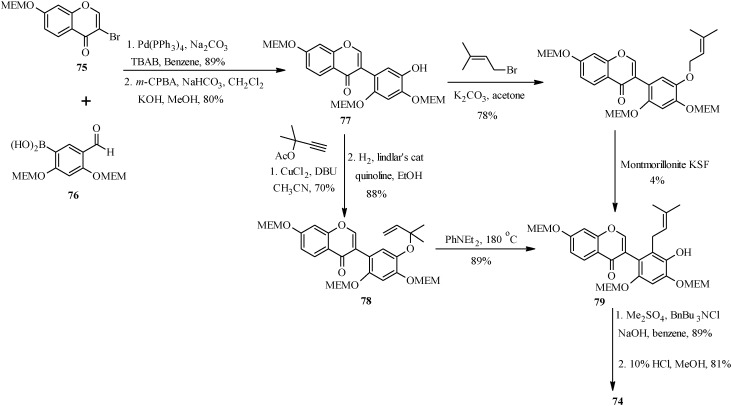
Total synthesis of kwakhurin (**74**).

As shown in Scheme 23, the first steps in the alternative route were preparation of the MEM-protected 3-bromochromone **75** and boronic acid **76**. These were coupled using Pd(PPh_3_)_4_ and TBAB in benzene and the formyl group was converted into a hydroxy group by Baeyer-Villiger oxidation and alkaline hydrolysis to give isoflavone **77** [1]. The prenyl group was introduced on the isoflavone **77** via two pathways. The first one involved *O*-prenylation and montmorillonite KSF-assisted 1,3-migration of the prenyl group. This gave the expected *C*-prenylated isoflavone **79** in a poor yield of 4%. The *C*-prenylated isoflavone **79** was obtained in a good yield upon *O*-propargylation of **77** followed by reduction of the propargyl ether and thermal rearrangement of the resulting 1,1-dimethylallyl ether **78**. The last steps were methylation of the 5'-hydroxy group and deprotection of the 2'- and 4'-hydroxy groups under acid conditions to give kwakhurin (**74**) [1].

**Figure 7:**
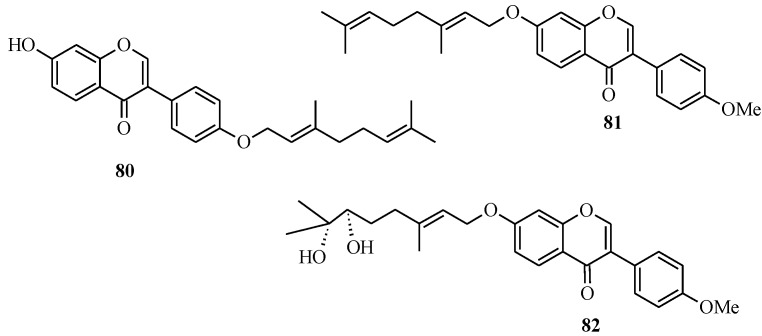
Structures of geranylated isoflavones.

In 2005, Felpin and co-workers developed a method for the Pd(C)-catalyzed Suzuki-Miyaura cross-coupling of iodocycloenones with arylboronic acids [11]. The use of the heterogeneous Pd(C) presents many advantages compared to other palladium-based catalysts. The reaction is conducted in the absence of additives and ligands, which are often expensive, and air and moisture sensitive. Furthermore, the catalyst can be easily removed from the reaction mixture by filtration, can be reused and is also compatible with water based solvents [11]. Felpin *et al.* further demonstrated the versatility of the Pd(C) chemistry in C-C bond forming reactions that led to the synthesis of geranylated isoflavones conrauinone D (**80**), 7-*O*-geranylformononentin (**81**) and griffonianone D (**82**) [12] (Figure 7). 

**Scheme 24:**
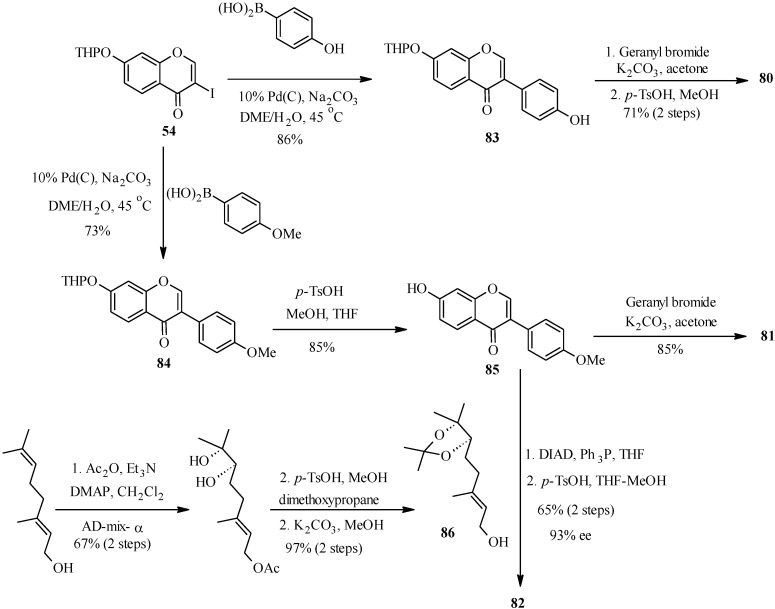
Synthesis of conrauinone D (**80**), 7-*O*-geranylformononentin (**81**) and griffonianone D (**82**) by Felpin *et al.* [12].

As illustrated in Scheme 24 [12], Pd(C)-catalyzed reaction of THP protected 3-iodochromone **54** with an 4-hydroxyphenylboronic acid or 4-methoxyphenylboronic acid gave isoflavone derivatives which could be used as precursors for the synthesis of the targeted compounds. 

Thus, conrauinone D (**80**) was prepared by *O*-alkylation of **83** with geranyl bromide using K_2_CO_3_ as base, followed by cleavage of the THP protecting group under mild conditions. *O*-Geranylation of the isoflavone **85**, obtained by the THP-deprotection of **84** gave 7-*O*-geranylformononentin (**81**). The last target compound griffonianone D (**82**) was prepared by Mitsunobu reaction of dihydroxylated geraniol derivative **86** and subsequent cleavage of the acetonide [12].

**Scheme 25:**
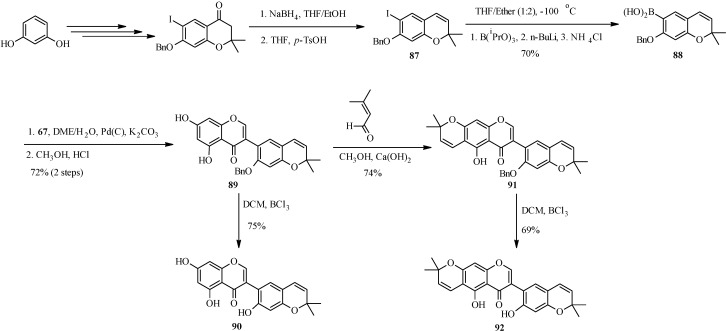
Total synthesis of kraussianone 1 (**92**) and eriosemaone D (**90**) [2].

The first total syntheses two biologically-active pyranoisoflavones, the anti-impotence pyranoisoflavone kraussianone 1 (**92**) and the anti-fungal pyranoisoflavone eriosemaone D (**90**) was reported by Selepe *et al.* in 2010 [2]. The key steps involved the Suzuki-Miyaura reaction for the construction of the isoflavone core and the regioselective formation of the dimethylpyran scaffolds to the phloroglucinol (A-ring) and resorcinol (B-ring) moieties (Scheme 25). The synthesis commenced by preparing the boronic acid **88** from aryl iodide **87** by an “*in situ* quench” procedure described for boronic acid **70**. Coupling of boronic acid **88** with 3-iodochromone **67** under Felpin’s conditions and subsequent removal of the MOM protecting groups gave a pyranoisoflavone **89**, which was transformed into eriosemaone D (**90**) by debenzylation with BCl_3_. Kraussianone 1 (**92**) was prepared by base-catalyzed aldol-type condensation of **91** with prenal followed by removal of the benzyl protecting group with BCl_3_ (Scheme 25) [2].

**Scheme 26:**
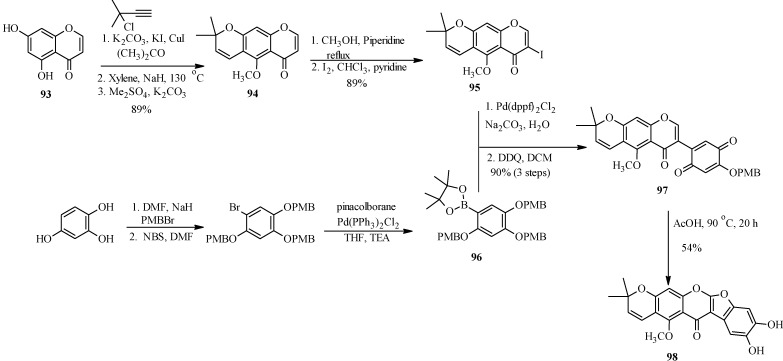
Total synthesis of hirtellanine A (**98**) [3].

The application of Suzuki-Miyaura reaction has also been demonstrated in the synthesis of other subclasses of isoflavonoids that include coumaronochromones. Zheng and Shen reported the total synthesis of hirtellanine A (**98**) [3], a coumaronochromone derivative that exhibits immunosuppresive activity (Scheme 26) [75]. The main precursors were 3-iodochromone bearing dimethylchromene scaffold **95** and a boronic ester **96**. The 3-iodochromone **95** was prepared in a sequence of steps, which involved regioselective formation the chromene scaffold by a Thom-Harfenist [76] rearrangement of the propargyl ether [77], prepared by *O*-alkylation of the 7-hydroxy group of chromone **93** with 3-chloro-3-methylbut-1-yne. Iodination of the resulting pyranochromone **94** at C-3 following Gammill’s protocol rendered **95**. The boronic ester **96** on the other hand was prepared in three steps from 1,2,4-trihydroxybenzene. The Suzuki coupling of **95** with **96** and subsequent oxidative cleavage of the *p*-methoxybenzyl protecting groups gave a quinone **97**, which upon treatment with acetic acid gave hirtellanine A (**98**) [3].

**Scheme 27:**
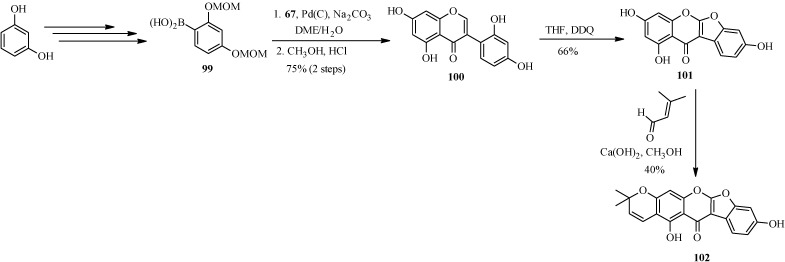
Total synthesis of lupinalbin H (**102**) [72].

Another pyranocoumaronochromone lupinalbin H (**102**) was synthesized by Selepe and co-workers in steps that involved preparation of 2'-hydroxygenistein (**100**) from 3-iodochromone **67** and a boronic acid **99**, followed by cyclodehydrogenation to lupinalbin A (**101**) [72]. The final step was the regioselective introduction of the dimethylpyran moiety to the A-ring of 101 via an aldol-type condensation with 3-methyl-2-butenal and 6π-electrocyclization (Scheme 27) [72].

## 5. Neoflavones

### 5.1. Synthesis of Neoflavones

The neoflavones (4-arylcoumarins) constitute the largest subclass of neoflavonoids. Traditional procedures for the preparation of neoflavones include the Pechmann or Perkin reactions and the Kostanecki acylation of 2-hydroxybenzophenones followed by base-catalyzed ring closure [77]. Other methods that have been developed include the Wittig reaction of 2-benzophenones [77] and metal-catalyzed cross-coupling reactions such as the Negishi-type, Stille-type and Suzuki-type reactions [8,9,77,78,79,80]. More recently, 4-arylcoumarins have been synthesized by direct arylation by the palladium-catalyzed oxidative Heck coupling of arylboronic acids to coumarins [81,82].

**Scheme 28:**
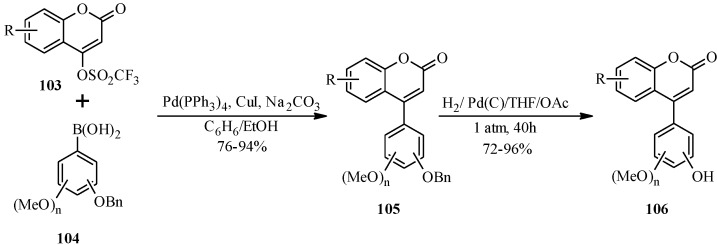
Synthesis of hydroxylated neoflavones by Donnelly and co-workers [84].

The synthesis of neoflavones by the Suzuki reaction was first reported by Donnelly and co-workers in 1996 [83]. Their approach involved coupling of 4-trifluoromethanesulfonyloxycoumarins with arylboronic acids in the presence of Pd(PPh_3_)_4_ and copper(1) iodide as a co-catalyst. The same research group later extended the procedure to the synthesis of hydroxylated neoflavones from benzyloxyboronic acids **104** and 4-trifluoromethanesulfonyloxycoumarins **103** as shown in Scheme 28 [84]. The benzyl protecting group was removed in the late stage by hydrogenation of **105** in the presence of Pd(C) in THF and AcOH to give **106**.

Subsequently, similar conditions have been applied in several instances in the syntheses of a series of neoflavones for the investigation of their biological activities [7,8,10]. For instance, neoflavones with a substitution pattern similar to the combretastatins have been prepared and evaluated for pharmacological activities that include, amongst others, cytotoxicity against CEM leukemia and HBL 100 epithelium cell lines [8,10] and antiprotozoal activity against *Plasmodium falciparum* and *Leishmania donovani* [7].

### 5.2. Synthesis of Arylated Neoflavones

**Scheme 29:**
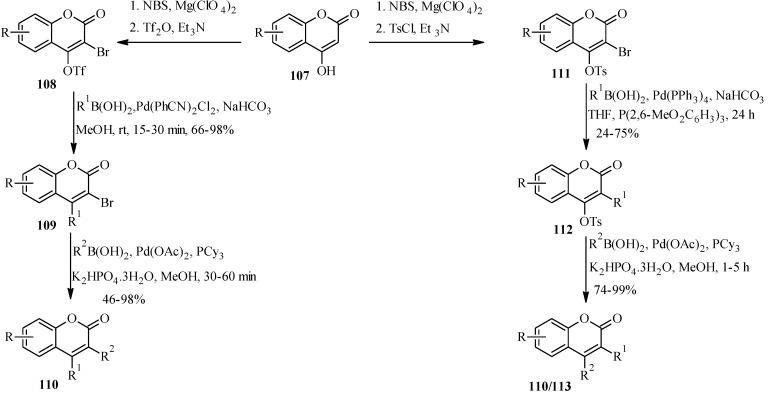
Synthesis of 3-arylcoumarins [79].

Arylated derivatives of neoflavones have been conveniently prepared by the palladium-catalyzed chemoselective cross-coupling of coumarins **107** activated at the different positions [79,80]. Zhang and colleagues prepared 3-arylneoflavones by a stepwise coupling of 3-bromo-4-trifloxycoumarin **108** or 3-bromo-4-tosyloxycoumarin **111** with differently substituted boronic acids (Scheme 29) [79].

Several palladium catalysts were screened under different conditions to determine the optimal conditions for the preparation of the bisarylated coumarins **110** and **113**. The order of reactivity of the active sites was found to be 4-OTf > 3-Br > 4-OTs [79]. Thus, **108** was monoarylated at C-4 under optimized conditions [Pd(PhCN)_2_Cl_2 _(5 mol%), 1 M NaHCO_3_, MeOH, rt, 15–30 min] to give 3-bromoneoflavones 109 in good yields. Subsequent coupling of 3-bromoneoflavones **109** with arylboronic acids using Pd(OAc)_2_ in the presence of PCy_3_ and K_2_HPO_4_.3H_2_O in MeOH gave 3-arylneoflavones **110**. In contrast, the 3-bromo-4-tosyloxycoumarin **111** reacted in the reversed order to give **112**, whereby the 3-bromo substituent was found to be more reactive than the tosyloxy group. An attempt to prepare the heteroarylcoumarins from the intermediates **108** and **111** in a one-pot sequential procedure gave the diarylcoumarins in low yields [79].

**Table:** 

R^1^	R^2^	Product
4-MeOC_6_H_4_	4-MeOC_6_H_4_	**110a**
4-MeOC_6_H_4_	4-CF_3_OC_6_H_4_	**110b**
4-MeOC_6_H_4_	4-MeC_6_H_4_	**110c**
4-MeOC_6_H_4_	C_6_H_5_	**110d**
4-MeOC_6_H_4_	4-NO_2_C_6_H_4_	**110e**
4-MeOC_6_H_4_	4-MeO_2_CC_6_H_4_	**110f**
C_6_H_5_	C_6_H_5_	**110g**
C_6_H_5_	4-MeOC_6_H_4_	**110h**
4-NO_2_C_6_H_4_	4-MeOC_6_H_4_	**110i**
4-MeO_2_CC_6_H_4_	4-MeOC_6_H_4_	**110j**
4-MeOC_6_H_4_	4-MeOC_6_H_4_	**110a**
4-MeOC_6_H_4_	4-CF_3_OC_6_H_4_	**113a**
4-MeOC_6_H_4_	4-MeC_6_H_4_	**113b**
4-MeOC_6_H_4_	C_6_H_5_	**110h**
4-MeOC_6_H_4_	4-MeO_2_CC_4_H_6_	**110j**
4-MeC_6_H_4_	4-MeC_6_H_4_	**113c**
4-MeC_6_H_4_	4-MeOC_6_H_4_	**110c**

**Scheme 30:**
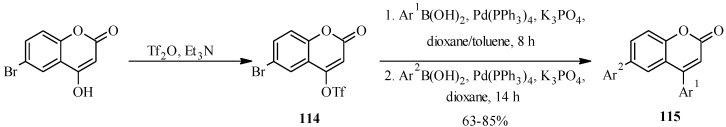
One-pot synthesis of 6-arylneoflavones from coumarin **114**.

More recently, Akrawi and co-workers chemoselectively prepared 6-arylated neoflavones **115a**–**e** by the reaction of 6-bromo-4-trifluoromethylsulfonyloxycoumarin (**114**) with arylboronic acids in the presence of Pd(PPh_3_)_4_ and K_3_PO_4_ as a base using a mixture of toluene/dioxane as solvent (Scheme 30) [80]. The reaction proceeded preferentially at C-4. This enabled a sequential one-pot reaction of **14** with two differently substituted phenylboronic acids to give heteroarylated compounds **115a**–**e** in good yields (Scheme 30). 

**Table:** 

115	Ar^1^	Ar^2^
**a**	4-MeOC_6_H_4_	4-ClC_6_H_4_
**b**	4-MeOC_6_H_4_	3-ClC_6_H_4_
**c**	3,5-Me_2_C_6_H_3_	4-MeOC_6_H_4_
**d**	4-ClC_6_H_4_	4-MeOC_6_H_4_
**e**	4-ClC_6_H_4_	3-FC_6_H_4_

## 6. Conclusions

The Suzuki-Miyaura reaction has been successfully applied in the synthesis of a variety of flavonoids. The main advantage of this reaction is the mild reaction conditions which are compatible with different functional groups and thus allow the synthesis of flavonoids containing a number of sensitive substituents.
